# Observing the Observer (I): Meta-Bayesian Models of Learning and Decision-Making

**DOI:** 10.1371/journal.pone.0015554

**Published:** 2010-12-14

**Authors:** Jean Daunizeau, Hanneke E. M. den Ouden, Matthias Pessiglione, Stefan J. Kiebel, Klaas E. Stephan, Karl J. Friston

**Affiliations:** 1 Wellcome Trust Centre for Neuroimaging, University College of London, London, United Kingdom; 2 Brain and Spine Institute, Hôpital Pitié-Salpêtrière, Paris, France; 3 Laboratory for Social and Neural Systems Research, Institute of Empirical Research in Economics, University of Zurich, Zurich, Switzerland; 4 Max Planck Institute for Human Cognitive and Brain Sciences, Leipzig, Germany; 5 Donders Institute for Brain, Cognition and Behaviour, Nijmegen, The Netherlands; Indiana University, United States of America

## Abstract

In this paper, we present a generic approach that can be used to infer how subjects make optimal decisions under uncertainty. This approach induces a distinction between a subject's *perceptual* model, which underlies the representation of a hidden “state of affairs” and a *response* model, which predicts the ensuing behavioural (or neurophysiological) responses to those inputs. We start with the premise that subjects continuously update a probabilistic representation of the causes of their sensory inputs to optimise their behaviour. In addition, subjects have preferences or goals that guide decisions about actions given the above uncertain representation of these hidden causes or state of affairs. From a Bayesian decision theoretic perspective, uncertain representations are so-called “posterior” beliefs, which are influenced by subjective “prior” beliefs. Preferences and goals are encoded through a “loss” (or “utility”) function, which measures the cost incurred by making any admissible decision for any given (hidden) state of affair. By assuming that subjects make optimal decisions on the basis of updated (posterior) beliefs and utility (loss) functions, one can evaluate the likelihood of observed behaviour. Critically, this enables one to “*observe the observer*”, i.e. identify (context- or subject-dependent) prior beliefs and utility-functions using psychophysical or neurophysiological measures. In this paper, we describe the main theoretical components of this *meta-Bayesian* approach (i.e. a Bayesian treatment of Bayesian decision theoretic predictions). In a companion paper (‘Observing the observer (II): deciding when to decide’), we describe a concrete implementation of it and demonstrate its utility by applying it to simulated and real reaction time data from an associative learning task.

## Introduction

This paper is about making inferences based on behavioural data in decision-making experiments. Unlike the analysis of most other types of data, behavioural responses made by subjects are themselves based on (perceptual) inferences. This means we have the special problem of making inferences about inferences (i.e., meta-inference). The basic idea we pursue is to embed perceptual inference in a generative model of decision-making that enables us, as experimenters, to infer the probabilistic representation of sensory contingencies and outcomes used by subjects. In one sense this is trivial; in that economic and computational models of decision-making have been optimized for decades, particularly in behavioural economics and neuroimaging (e.g. [1]–[3]). However, we address the slightly deeper problem of how to incorporate subjects' inferences *per se*. This speaks to a growing interest in how the brain represents uncertainty (e.g., probabilistic neuronal codes ([4]) and acts as an inference machine ([5]–[7]). Furthermore, we are interested in a general framework that can be adapted to most experimental paradigms. We hope to show that suitably formulated models of perception and decision-making enable inference on subjective beliefs, even when using data as simple as reaction times. In a companion paper (‘Observing the observer (II): deciding when to decide’, we illustrate the approach using reaction times to make inferences about the prior beliefs subjects bring to associative learning tasks and how these are expressed behaviourally in the context of speed-accuracy trade-offs.

One may wonder: why the emphasis on perceptual inference? We live in a world of uncertainty and this has led many to suggest that probabilistic inference may be useful for describing how the brain represents the world and optimises its decisions (e.g. [8] or [9]). A growing body of psychophysical evidence suggests that we behave as Bayesian observers; i.e. that we represent the causes of sensory inputs by combining prior beliefs and sensory information in a Bayes optimal fashion. This is manifest at many temporal and processing scales; *e.g.*, low-level visual processing ([10]–[16]), multimodal sensory integration ([17]–[21]), sensorimotor learning ([22]–[27]), conditioning in a volatile environment ([28]–[29]), attention ([30]–[31]), and even reasoning ([32]–[33]). This *Bayesian observer* assumption provides principled constraints on the computations that may underlie perceptual inference, learning and decision-making.

In order to describe behavioural responses within a Bayesian decision theoretic framework (see *e.g.* [34]) one has to consider two levels of inference. Firstly, at the *subject level*: a Bayesian subject or observer relies on a set of prior assumptions how sensory inputs are caused by the environment. In the following, we will call this mapping, from environmental causes to sensory inputs, the *perceptual model*. Secondly, at the experimenter level: as we observe the observer, we measure the consequences of their posterior belief about sensory cues. In the following, we will call this mapping, from sensory cues to observed responses, the response model. Crucially, the response model subsumes the perceptual model because the perceptual model determines the subject's beliefs and responses. This means inverting the response model (to map from responses to their causes) necessarily incorporates an inversion of the perceptual model (to map from sensory cues to the beliefs that cause those responses). When measuring explicit actions (i.e., the subject's decisions), the response model also invokes utility- or loss-functions, which encode the subject's goals and preferences.

**Figure 1:**
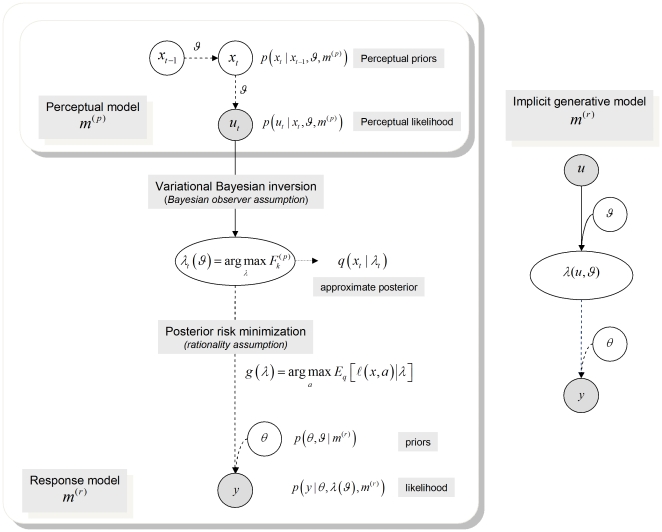
Conditional dependencies in perceptual and response models. The lines indicate conditional dependence among the variables in each model (broken lines indicate probabilistic dependencies and solid lines indicate deterministic dependencies). **Left**: perceptual and response models. **Right**: Implicit generative model, where the perceptual model is assumed to be inverted under ideal Bayesian assumptions to provide a mapping (through recognition) from sensory input to observed subject responses.

The perceptual model predicts the subject's sensory signals (i.e. inputs arising from environmental causes), and the response model predicts the subject's responses in terms of behaviour (e.g. decisions or reaction times) and/or neurophysiology (e.g. brain activity). For example, in the context of an associative learning paradigm, the unknown quantities in the perceptual model are causal associations among stimuli; whereas the unknown variables in the response model are the brain's representations of these associations (*i.e*. the brain states that encode posterior beliefs) and/or the ensuing behavioural responses (depending on which measurements are available). Critically, the response model subsumes the perceptual model, how it is inverted and how the ensuing posterior belief maps to measurable responses (Figure 1).

The distinction between these two models is important. In perceptual learning studies, the experimenter is interested in both the perceptual model and the mechanics of its inversion. For example, the computational processes underlying low-level vision may rest on priors that finesse ambiguous perception (e.g. [16]). The relevant variables (e.g. those encoding prior beliefs) are hidden and can only be inferred through experimental observations using a response model. Conversely, in decision-making studies, the experimenter is usually interested in the response model, because it embodies the utility- or loss-functions and policies employed by the subject (e.g., see [26] for an application to the motor system). Note that the response model may (implicitly) subsume the subject's perceptual model of the world, under which expected utility is evaluated. This dependency induces the Inverse Bayesian Decision Theoretic (IBDT) problem: to determine a subject's prior beliefs and goals (i.e. loss-function), given their observed behaviour to known sensory inputs.

The complete class theorem states that any admissible decision rule is Bayes-optimal for at least one set of prior beliefs and loss-function [34]. This means that there is always a solution to the IBDT problem. It is also known from game theory that many combinations of beliefs and preferences are consistent with the same behaviour [35]. In other words, the solution to the IBDT problem exists but *is not* unique; i.e. the problem is under-determined or ill-posed [36]. This has led researchers to focus on restricted classes of the general IBDT problem. These schemes differ in terms of the constraints that are used to overcome its indeterminacy; for example, inverse decision theory ([37], [26]), inverse game theory ([38]), inverse reinforcement learning ([39]–[41]) or inverse optimal control ([42]). However, these schemes are not optimally suited for the kind of experiments commonly used in neuroscience, behavioural economics or experimental psychology, which usually entail partial knowledge about the beliefs and losses that might underlie observed behaviour.

This paper proposes an approximate solution to the IBDT problem for these types of experimental paradigms. The approach derives from a variational Bayesian perspective ([43]), which is used both at the subject level (to model perceptual inference or learning) and at the experimenter level (to model behavioural observations). The approach allows one to estimate model parameters and compare alternative models of perception and decision-making in terms of their evidence. We will first recall the IBDT problem and then describe the basic elements of the framework. Finally, we will discuss the nature of this meta-Bayesian approach. A practical implementation of it is demonstrated in a companion paper (‘Observing the observer (II): deciding when to decide’).

## Methods

In this section, we present the basic elements of the framework. We first recall the prerequisites of Bayesian Decision Theory as generically and simply as possible. We then describe the form of perceptual models and their (variational) Bayesian inversion. This inversion provides an implicit mapping from cues to internal representations and describes recognition under the Bayesian observer assumption. We then consider response models for behaviourally observed decisions, which subsume Bayes optimal recognition. Finally, we will cover the inversion of response models, which furnishes an approximate (variational) Bayesian solution to the IBDT problem.

### Inverse Bayesian Decision Theory (IBDT)

We start with a perceptual model 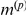 that specifies the subject's probabilistic assumptions about how sensory inputs are generated. Sensory inputs 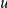 (experimental stimuli) are generated from hidden causes 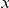 (experimental factors or states) and are expressed in terms of two probability density functions: the observer's (subject's) likelihood function 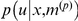 and prior beliefs about hidden states of the world 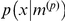. In the following, we will use “hidden causes”, “environmental states” or “states of affairs” as interchangeable terms. The hidden states are unknown to the subject but might be under experimental control. For example, in the context of associative learning, sensory information 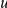 could consist of trial-wise cue-outcome pairings, and 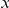 might encode the probabilistic association between cues and outcomes that is hidden and has to be learnt.

The subject's likelihood quantifies the probability of sensory input given its hidden cause. The priors encode the subject's belief about the hidden states before any observations are made. The likelihood and priors are combined to provide a probabilistic model of the world:
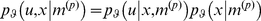
(1)where we have used the notation 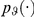 to indicate a parameterization of the likelihood and priors by some variables 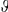. These *perceptual parameters* encode assumptions about how states and sensory inputs are generated. We assume that 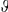 have been optimised by the subject (during ontogeny) but are unknown to the experimenter.

Bayesian inversion of this perceptual model corresponds to *recognising* states generating sensory input and learning their causal structure. This recognition is encoded by the subject's posterior density; 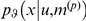, which obtains from Bayes' rule:
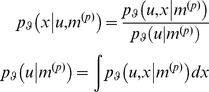
(2)


Here, 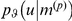 is the marginal likelihood of sensory inputs 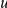 under the perceptual model 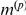, i.e. the (perceptual) model evidence (where the perceptual parameters 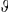 have been integrated out). Bayes' rule allows the subject to update beliefs over hidden states from the prior 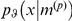 to the posterior 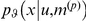 on the basis of sensory information (encoded by the likelihood 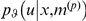). Since the posterior represents information about the hidden states given some sensory inputs, we will refer to it as a *representation*.

We can describe *recognition* as a mapping from past sensory inputs 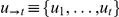 to the current representation: 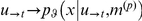, where 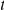 indexes time or trial. The form of Equations (1) and (2) mean that representations 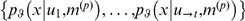 form a Markovian sequence, where 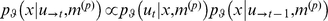. In other words, the current belief depends only upon past beliefs and current sensory input.

Subjects' responses may be of a neurophysiological and/or behavioural nature and may reflect perceptual representations or explicit decisions. In the latter case, we need to model the mapping from representations to action, 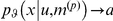, which we call the *response model*. This entails specifying the mechanisms through which representations are used to form decisions. Within a Bayesian decision theoretic (BDT) framework, policies rely on some form of rationality. Under rationality assumptions, the subject's policy (i.e. decision) is determined by a *loss-function*, 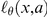, which returns the cost incurred by taking action 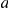 while the state of affairs is 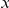. The loss-function is specified by some parameters 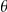 that are unknown to the experimenter. In the economics and reinforcement learning literature one usually refers to *utility*, which is simply negative loss. BDT gives the rational policy, under uncertainty about environmental states, in terms of the optimal action 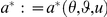 that minimizes *posterior risk* 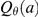; i.e. expected loss:
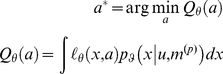
(3)where the expectation is with regard to the posterior density on the hidden states. This renders optimal decisions dependent upon both the perceptual model 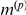 and the loss-function 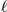.

The complete-class theorem ([34]) states that any given policy or decision-rule is optimal for at least one pairing of model and loss-function 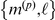. Crucially, this pair is never unique; i.e. the respective contribution of the two cannot be identified uniquely from observed behaviour. This means that the inverse Bayesian decision theoretic (IBDT) problem is ill-posed in a maximum likelihood sense. Even when restricted to inference on the loss-function (i.e., when treating the perceptual model as known) it can be difficult to solve (e.g., see [39] or [40]). This is partly because solving Equations 2 and 3 is analytically intractable for most realistic perceptual models and loss-functions. However, this does not mean that estimating the parameters 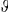 and 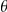 from observed behaviour is necessarily ill-posed: if prior knowledge about the structure of the perceptual and response models is available we can place priors on the parameters. The ensuing regularisation facilitates finding a unique solution.

In the following, we describe an approximate solution based upon a variational Bayesian formulation of perceptual recognition. This allows us to find an approximate solution to Equation 2 and simplify the IBDT problem for inference on subject-specific cognitive representations that underlie behaviourally observed responses.

### Variational treatment of the perceptual model

Variational Bayesian inference furnishes an approximate posterior density on the hidden states 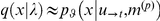, which we assume to be encoded by some variables 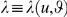 in the brain. These are the sufficient statistics (*e.g*., mean and variance) of the subject's posterior belief. They encode the subject's representation and depend on sensory inputs and parameters of the perceptual model. Recognition now becomes the mapping from sensory inputs to the sufficient statistics 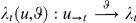. We will refer to 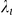 as the representation at time (or trial) 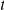. In variational schemes, Bayes' rule is implemented by optimising a free-energy bound 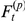 on the log-evidence for a model, where by Jensen's inequality
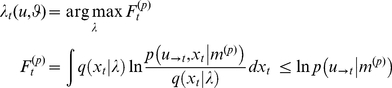
(4)


Maximizing the perceptual free-energy 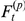 minimizes the Kullback-Leibler divergence between the exact 
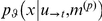
 and approximate 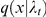 posteriors. Strictly speaking, the free-energy in this and subsequent equations of this paper should be called “negative free-energy” due to its correspondence to negative Helmholtz free-energy in statistical physics. For brevity, we will only refer to “free-energy” throughout the paper and omit “negative” when relating recognition and inference to maximisation of free-energy. Under some simplifying assumptions about the approximate posterior, this optimization is much easier than the integration required by Bayes' rule (Equation 2). Appendix S1 of this document summarizes the typical (e.g. Laplace) approximations that are required to derive such an approximate but analytical inversion of any generative model. In short, within a variational Bayesian framework, recognition can be reduced to optimizing the (free-energy) bound on the log-evidence with respect to the sufficient statistics 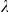 of the approximate posterior (e.g., first and second order moments of the density).

As a final note on the perceptual model, it is worth pointing out that recognition, i.e. the sequence of representations, 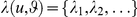, has an explicit Markovian form:
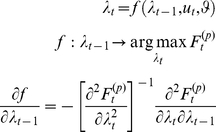
(5)where the evolution function 
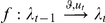
 is analytical and depends on the perceptual model through the perceptual free energy. Note that the last line of equation 5 is obtained with the use of implicit derivatives. In summary, recognition can be formulated as a finite-dimensional analytical state-space model (c.f. Equation 5), which, as shown below, affords a significant simplification of the IBDT problem. Note that under the Laplace approximation (see Appendix S1), the sufficient statistics 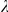 are simply the mode of the approximate posterior, and the gradient of the evolution function w.r.t. 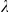 writes:
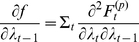
(6)where 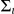 is the second-order moment of the approximate posterior (the covariance matrix), which measures how uncertain are subjects about the object of recognition. This is important since it means that learning effects (i.e. the influence of the previous representation onto the current one) are linearly proportional to perceptual uncertainty (as measured by the posterior variance).

### The response model

To make inferences about the subject's perceptual model we need to embed it in a generative model of the subject's responses. This is because for the experimenter the perceptual representations are hidden states; they can only be inferred through the measured physiological or behavioural responses 
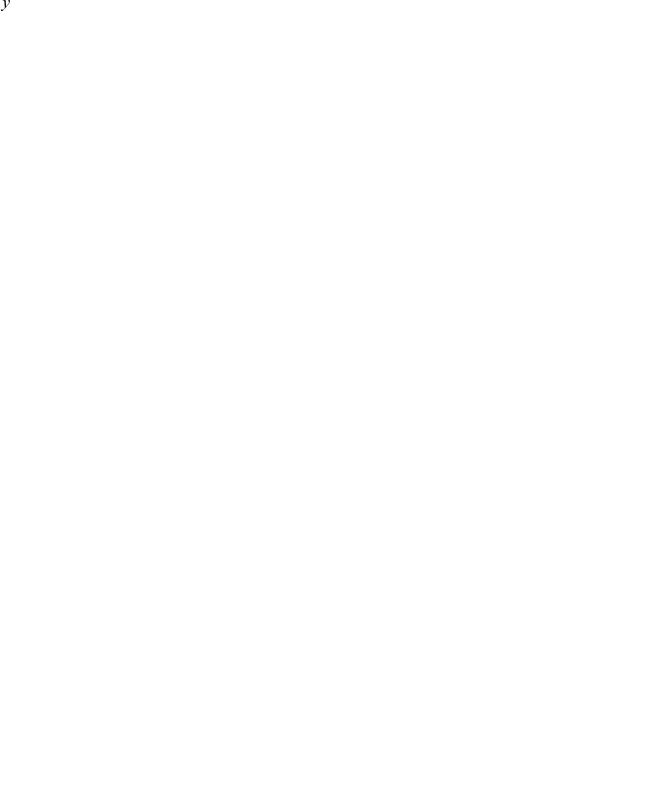
 that they cause. The response model 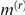 can be specified in terms of its likelihood (first equation) and priors (second equation)
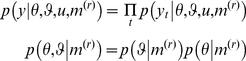
(7)


Note that the observed trial-wise responses 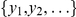 are conditionally independent, given the current representation. The unknown parameters 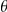 of the response model determine how the subject's representations are expressed in measured responses (and include the parameters of any loss-function used to optimise the response; see below). The priors 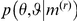 cover the parameters of both the response and perceptual models. The dependence of the response model 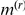 upon the perceptual model 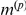 is implicit in the form of the recognition process 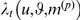.

This paper deals with neuroscientific measurements of physiological or behavioural responses. For this class of responses, the form of the likelihood of the response model can be described by a mapping 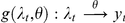 from the representation to the measurement. For example, the response likelihood can be expressed as the state-space model
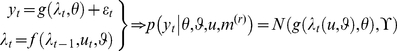
(8)where 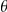 are response parameters that are required to specify the mapping and 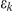 is a zero-mean Gaussian residual error with covariance 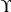. The evolution function 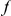 models the time (or trial) dependent recognition process (see Equation 5 above). The observation mapping 
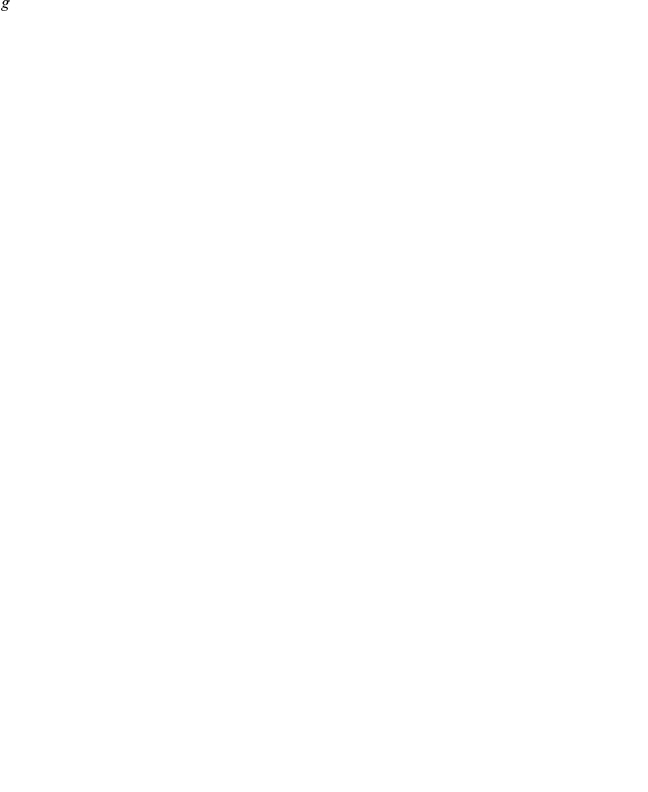
 could be a mapping between representations and neuronal activity as measured with EEG or fMRI (see e.g. [7]), or between representations and behavioural responses (e.g. [44]). In the context of IBDT, the measured response is an action or decision that depends on the representation. Rationality assumptions then provide a specific (and analytic) form for the mapping to observed behaviour
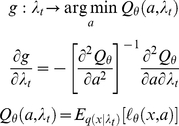
(9)where 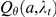 is the posterior risk (Equation 3). In economics and reinforcement learning decisions are sometimes considered as being perturbed by noise (see, e.g. [23]) that scales with the posterior risk of admissible decisions. The response likelihood that encodes the ensuing policy then typically takes the form of a logit or *softmax* (rather than *max*) function.

To invert the response model we need to specify the form of the loss-function 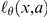 so that the subject's posterior risk 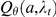 can be evaluated. We also need to specify the perceptual model 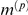 and the (variational Bayesian) inversion scheme that determine the subject's representations. Given the form of the perceptual model (that includes priors) and the loss-function (that encodes preferences and goals), the observed responses depend only on the perceptual parameters 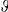 (that parameterize the mapping of sensory cues to brain states) and response parameters 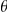 (that parameterize the mapping of brain states to responses). Having discussed the form of response models, we now turn to their Bayesian selection and inversion.

### Variational treatment of the response model

Having specified the response model in terms of its likelihood and priors, we can recover the unknown parameters describing the subject's prior belief and loss structure, using the same variational approach as for the perceptual model (see Appendix S1):
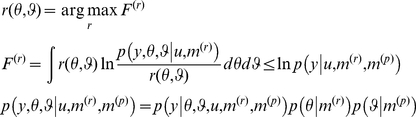
(10)


This furnishes an approximate posterior density 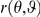 on the response and perceptual parameters.

Furthermore, we can use the free-energy 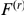 as a lower bound approximation to the log-evidence for the *i*-th perceptual model, under the *j*-th response model
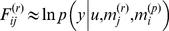
(11)


Note that 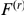 as an approximation to the evidence of the response model should not be confused with the *perceptual* free energy 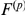 in Equation 4. This bound can be evaluated for all plausible pairs of perceptual and response models, which can then be compared in terms of their evidence in the usual way. Crucially, the free-energy 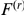 accounts for any differences in the complexity of the perceptual or response model [45]. Furthermore, this variational treatment allows us to estimate the perceptual parameters, which determine the sufficient statistics 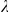 of the subject's representation. This means we can also estimate the subject's posterior belief, while accounting for our (the experimenter's) posterior uncertainty about the model parameters
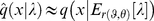
(12)where 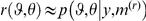 is the variational approximation to the marginal posterior of the perceptual parameters, obtained by inverting the response model (see Equation 10). In general, equation 12 means that our estimate of the subject's uncertainty may be “inflated” by our experimental uncertainty (c.f. equation 23 below and discussion section).

Lastly, the acute reader might have noticed that there is a link between the response free energy and the perceptual free energy. Under the Laplace approximation (see Appendix S1), it actually becomes possible to write the former as an analytical function of the latter:
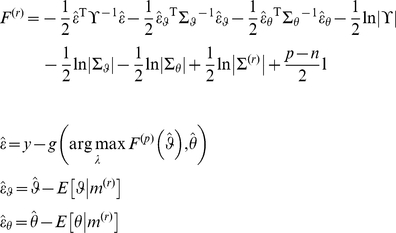
(13)where the response model is of the form given in equation 8 and we have both assumed that the residuals covariance 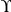 was known and dropped any time/trial index for simplicity. In equation 13, 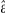 is the estimated model residuals and 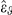 (respectively, 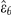) is the estimated deviations of the perceptual (respectively, response) parameters to their prior expectations 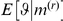 (respectively, 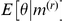), under the response model. These prior expectations (as well as any precision hyperparameters of the response model) can be chosen arbitrarily in order to inform the solution of the IBDT problem, or optimized in a hierarchical manner (see for example companion paper). Note that 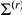 is the second-order moment (covariance matrix) of the approximate posterior 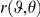 over the perceptual and response parameters (whose dimension is 
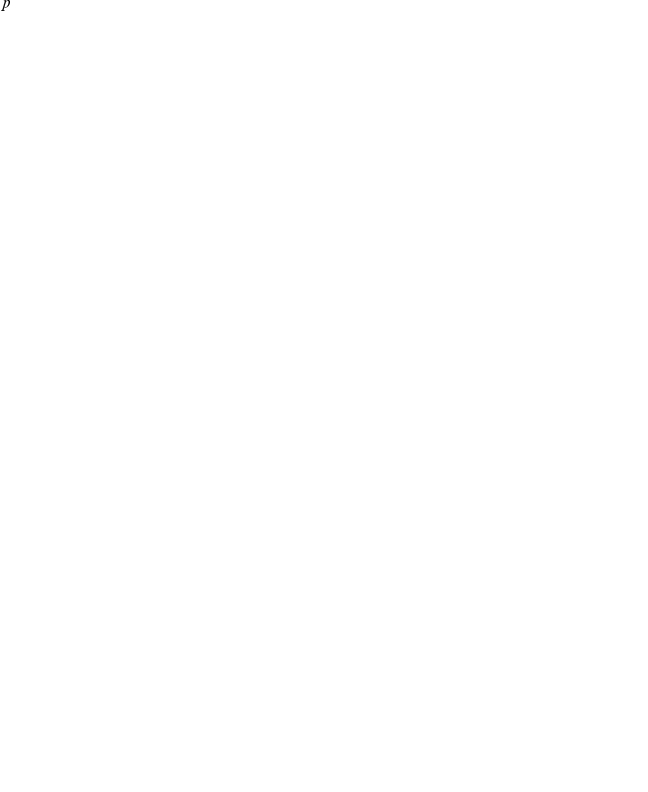
), 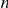 is the dimension of the data and 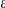 is the response model residuals (see equation 8). The posterior covariance 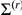 quantifies how well information about perceptual and response model parameters can be retrieved from the (behavioural) data:
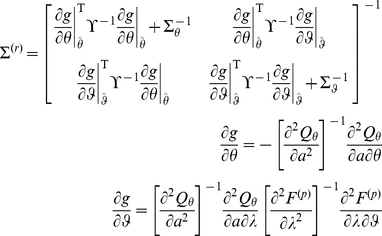
(14)where 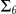 (respectively, 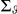) is the prior covariance matrix of the response (respectively, perceptual) parameters. Equation 14 is important, since it allows one to analyze potential non-identifiability issues, which would be expressed as strong non-diagonal elements in the posterior covariance matrix 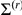.

It can be seen from equation 14 that, under the Laplace approximation, the second-order moment 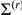 of the approximate posterior density 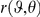 over perceptual and response model parameters is generally dependent upon its first-order moment 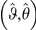. The latter, however, is simply found by minimizing a regularized sum-of-squared error:
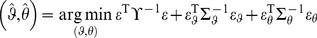
(15)


Note that equation 15 does not hold for inference on hyperparameters (e.g., residual variance 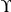). In this case, variational Bayesian under the Laplace approximation iterates between the optimization of parameters and hyperparameters, where the latter basically maximize a regularized quadratic approximation to Equation 13. We refer the interested reader to the Appendix S1 of this manuscript, as well as to [46].

## Results

### A simple perception example

Consider the following toy example: subjects are asked to identify the mean of a signal 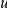 using the fewest samples of it as possible. We might consider that their perceptual model 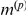 is of the following form:
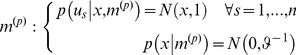
(16)where 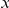 is the unknown mean of the signal 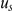, 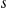 indexes the samples (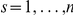), 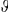 is the prior precision of the mean signal (unknown to us) and we have assumed that subjects know the (unitary) variance of the signal. In this example, 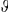 is our only perceptual parameter, which will be shown to modulate the subject's observed responses. The loss function of this task is a trade-off between accuracy and number of samples and could be written as:
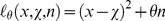
(17)where 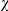 is the subject's estimator of the mean of the signal and 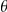 balances the accuracy term with the (linear) cost of sampling size 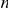. Subjects have to choose both a sampling size 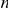 and an estimator 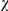 of the mean signal, which are partly determined by our response parameter 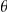. We now ask the question: what can we say about the subject's belief upon the signal mean, given its observed behaviour?

Under the perceptual model given in equation 16, it can be shown that the perceptual free energy, having observed 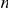 samples of the signal, has the following form:
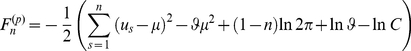
(18)where the optimal sufficient statistics 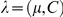 of the subject's (Gaussian) posterior density 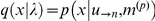 of the mean signal are given by:
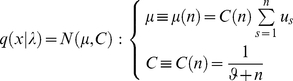
(19)


Equation 19 shows that the posterior precision grows linearly with the number of samples.

Under the loss function given in equation 17, it is trivial to show that the posterior risk, having observed 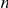 samples of the signal, can then be written as:
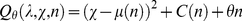
(20)


From equation 19, it can be seen that the optimal estimator of the mean signal is always equal to the its posterior mean, i.e.: 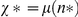, where 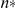 is the optimal sample size:
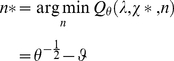
(21)


We consider that both the chosen mean signal estimator and the sample size are experimentally measured, i.e. the response model has the following form:
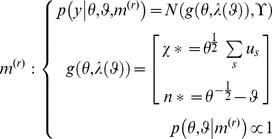
(22)where 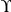 is the variance of the response model residuals, 
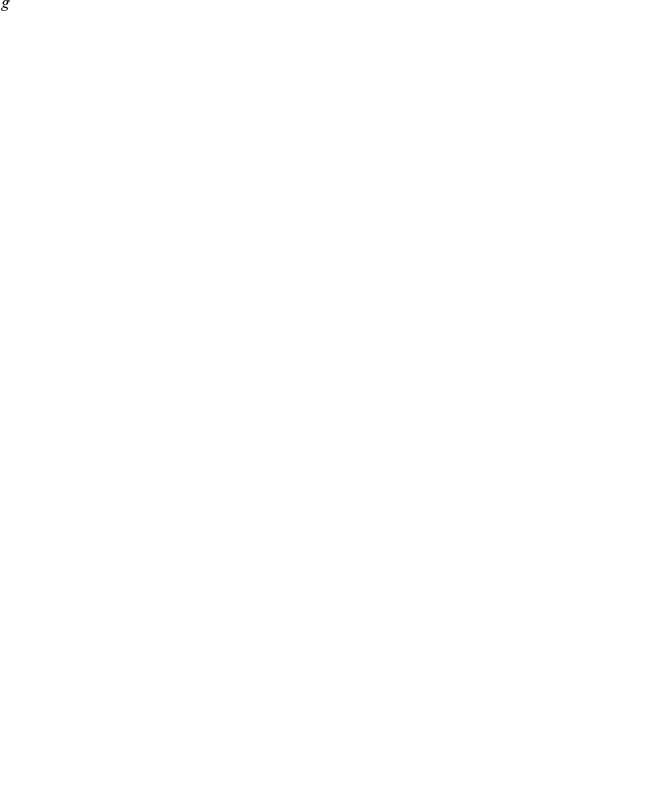
 is the mapping from the representation of the mean signal (as parameterized by the sufficient statistics 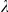) to the observed choices and we have used non informative priors on both perceptual and response parameters. Following equation 14, it can be shown that, under the Laplace approximation, the experimenter's posterior covariance on the perceptual and response parameters is given by:
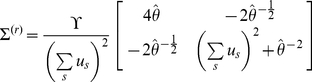
(23)where 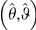 is the first-order moment of 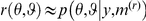, the approximate posterior density on the model parameters. These estimates are found by maximizing their variational energy (c.f. equation 15). The covariance matrix 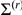 in equation 23 should not be confused with 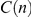 in equation 19, which is the second-order moment (variance) of the subject's posterior density over the mean signal 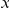. The latter is an explicit function of the prior precision 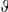 over the mean. The former measures the precision with which one can experimentally estimate 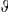, given behavioural measures 
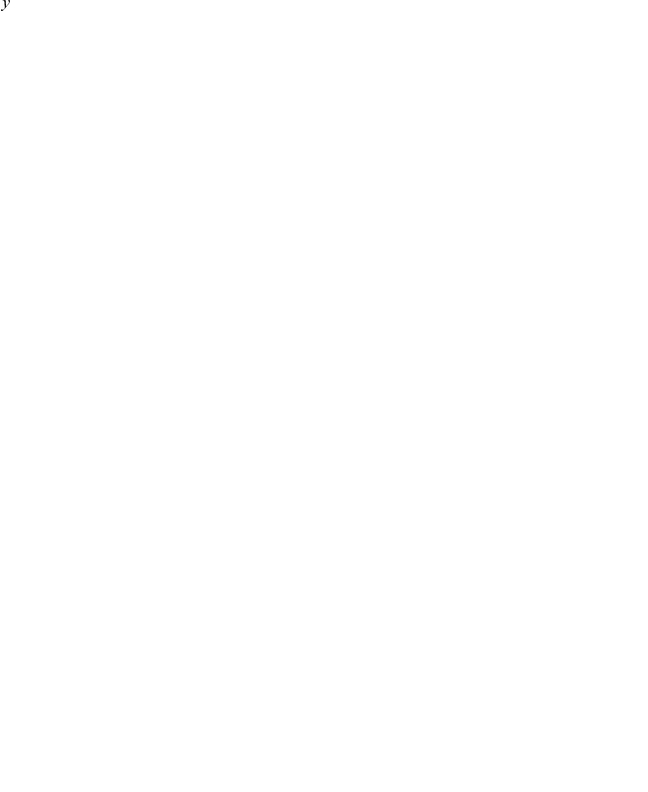
. Following equations 12 and 23, the experimenter estimate of the subject's belief about the signal mean can then be approximated (to first order) as:
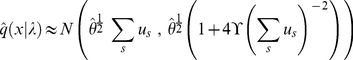
(24)


It can be seen from equation 24 that the variance 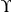 of the response model residuals linearly scales our estimate of the subject's uncertainty about the signal mean 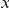. This is because we accounted for our (the experimenter's) uncertainty about the model parameters.

A number of additional comments can be made at this point.

First, the optimal sample size given in equation 21 can be related to evidence accumulation models (e.g. [47]–[49]). This is because the sample size 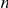 plays the role of artificial time in our example. As 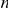 increases, the posterior variance 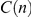 decreases (see equation 19) until it reaches a threshold that is determined by 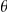. This threshold is such that the gain in evidence (as quantified by the decrease of 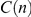) just compensates for the sample size cost 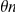. It should be noted that there would be no such optimal threshold if there was no cost to sensory sampling.

Second, it can be seen from equation 23 that our posterior uncertainty about the model (response and perceptual) parameters decreases with the power of the sensory signals 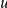. This means that, from an experimental design perspective, one might want to expose the subjects with sensory signals with high magnitude. More generally, the experimenter's posterior covariance matrix will always depend onto the sensory signals, through the recognition process. This means that it will always be possible to optimize the experimental design with respect to the sensory signals 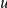, provided that a set of perceptual and response models are specified prior to the experiment.

### Summary

In summary, by assuming that subjects optimise a bound on the evidence or marginal likelihood 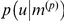 for their perceptual model, we can identify a sequence of unique brain states or representations 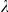 encoding their posterior beliefs 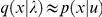. This representation, which is conditional upon a perceptual model 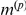, then enters a response model 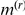 of measured behavioural responses 
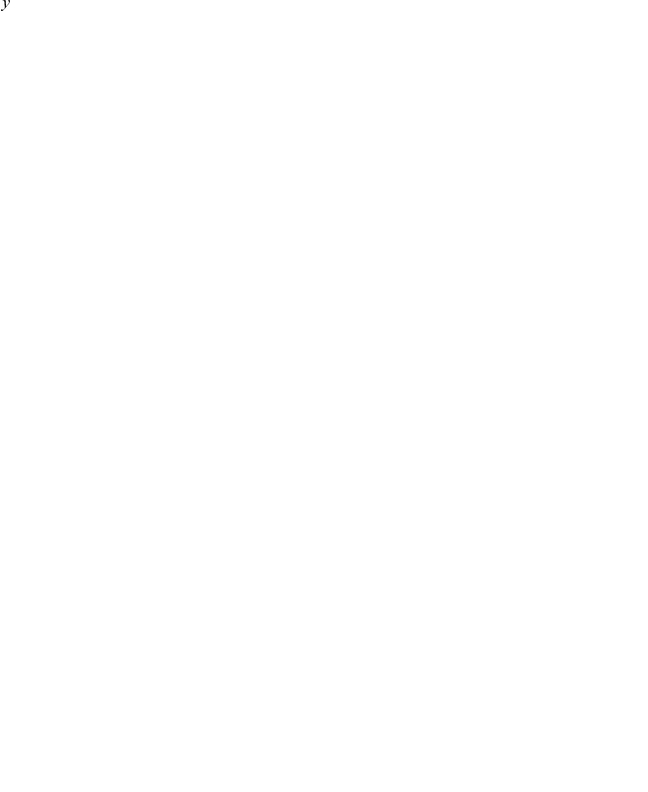
. This is summarised in Figure 1. Solving the IBDT problem, or *observing the observer*, then reduces to inverting the response model, given experimentally observed behaviour. This meta-Bayesian approach provides an approximate solution to the IBDT problem; in terms of model comparison for any combinations of perceptual and response models and inference on the parameters of those models. This is important, since comparing different perceptual models 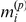 (respectively response models) in the light of behavioural responses means we can distinguish between qualitatively different prior beliefs (respectively utility/loss functions) that the subject might use. We illustrate this approach on an application to associative learning in a companion paper ‘*Observing the observer (II): deciding when to decide*’.

## Discussion

We have described a variational framework for solving the Inverse Bayesian Decision Theory (IBDT) problem in the context of perception, learning and decision-making. This rests on formulating a generative model of observed subject responses in terms of a perceptual-response model pair (Figure 1): Ideal Bayesian observer assumptions map experimental stimuli to perceptual representations, under a perceptual model; 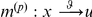; while representations determine subject responses, under a response model; 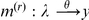. The central idea of our approach is to make inversion of the perceptual model (i.e. recognition: 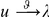) part of the response model. This provides a complete mapping 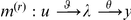 from experimental stimuli to observed responses.

We have used the term ‘meta-Bayesian’ to describe our approach because, as they *observe the observer*, experimenters make (Bayesian) statistical inferences about subject's (Bayesian) perceptual inferences (i.e., an inference about an inference). In other words, we solve the inverse problem of how subjects solve theirs. The subject's inverse problem is to recognize (estimate) the hidden causes of their sensory signals, under some prior assumptions (the perceptual model) about these causes. In contrast, the experimenter's inverse problem is to identify both the subject's prior beliefs (which influence their recognition process) and their preferences (which maps their recognition process to decisions expressed by observed actions). This is closely related to, but distinct from, ‘meta-cognition’, where subjects make inferences about their own inferences (for example, when rating one's confidence about a decision). Having said this, some forms of meta-cognition could be modelled using the proposed meta-Bayesian framework. For example, theory of mind [50]; i.e. the ability to identify the beliefs and intentions of others, could also be framed as solving the inverse problem of how others have solved theirs (see [51] for a discussion of related issues about bounded rationality in the context of game theory).

Note that the recognition process 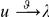 is expected to (strongly?) depend on the subject's priors about the hidden state of affairs, which is shaped by their previous sensory experiences. This means that we expect the subject's behaviour to vary according to their subjective prior beliefs. The latitude afforded by a dependence on priors is a consequence of optimal perception; and the nature of perceptual illusions has provided very useful insights into the structure of priors the brain might use (see e.g., [16] or [14]). These experiments can be thought of as having disclosed ‘effective’ (context-dependent) priors of the brain, in the sense that they revealed a specific aspect of the highly complex perceptual model that underlies the brain's perceptual and learning machinery. According to the complete class theorem (see e.g., [34]), there is always at least a set of priors and loss functions that can explain any observed behaviour. This means that one might not be able to experimentally refute the hypothesis that the brain acts as a Bayesian observer. However, one might be able to experimentally identify the effective priors of this virtual brain, which should prove useful in robustly predicting behavioural trends.

It is possible (in principle) to use the current framework with experimental measures of neurophysiological responses (c.f. section ‘The response model’). To do this, one would need to specify the response model in terms of how neural activity encodes subjective representations and how brain connectivity subtends recognition and learning. Such principles have already been proposed as part of the ‘Bayesian brain’ hypothesis (see, e.g., [7], [52], [53], [13]). In brief, the perceptual model is assumed to be hierarchically deployed in sensory cortex. Recognition is mapped onto this anatomical hierarchy through top-down predictions and bottom-up prediction error messages between different levels of a hierarchical perceptual model, to provide a Bayesian variant of predictive coding [6]. Note that these theories also consider the role of neuromodulators [54]–[55] and the nature of motor outputs; i.e. behavioural responses ([22], [56], [57], [58], [59]). However, there is an ongoing debate about the “site” of decision-making in the cortex (e.g. [60]) and so far no comprehensive theory exists that describes, in precise terms, the neural and computational substrates of high-level decisions and associated processes, such as the affective value of choices.

Experimental measures of decisions or choices deserve an additional comment. This is because in this case, care has to be taken with approximations to the optimal policy, when closed-form solutions are not available. This might be an acute problem in control theoretic problems, where actions influence the (hidden) states of the environment. In this case, the posterior risk becomes a function of action (which is itself a function of time). Minimizing the posterior risk then involves solving the famous Bellman equation (see e.g. [61]), which does not have closed-form solutions for non-trivial loss-functions. The situation is similar in game theory, when a subject's loss depends on the decisions of the other players. So far, game theory has mainly focussed on deriving equilibria (e.g. Pareto and Nash equilibria, see [62]), where the minimization of posterior risk can be a difficult problem. Nevertheless, for both control and game theoretical cases, a potential remedy for the lack of analytically tractable optimal policies could be to compare different (closed-form) approximations in terms of their model evidence, given observed decisions. Fortunately, there are many approximate solutions to the Bellman equation in the reinforcement learning literature; e.g.: dynamic programming, temporal difference learning and Q-learning ([63]–[65]).

The complete class theorem states that there is always a pair of prior and loss functions, for which observed decisions are optimal in a Bayesian sense. This means it is always possible to interpret observed behaviour within a BDT framework (i.e., there is always a solution to the IBDT problem). Having said this, the proposed framework could be adapted to deal with the treatment of non-Bayesian models of learning and decision making. For example, frequentist models could be employed, in which equation 3 would be replaced by a minimax decision rule: 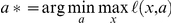. In this frequentist case, generic statistical assumptions about the response model residuals (see equations 7 and 8) would enable one to evaluate, as in the Bayesian case, the response model evidence (see equations 10 and 11). Since the comparison of any competing models (including Bayesian vs. non-Bayesian models) is valid whenever these models are compared in terms of their respective model evidence with regard to the same experimental data, our framework should support formal answers to questions about whether aspects of human learning and decision-making are of a non-Bayesian nature (cf. [66]).

Strictly speaking, there is no interaction between the perceptual and the response model, because the former is an attribute of the subject and the latter pertains to the (post hoc) analysis of behavioural data. However, this does not mean that neurophysiological or behavioural responses cannot feedback to the recognition process. For example, whenever the observer's responses influence the (evolution of the) state of the environment, this induces a change in sensory signals. This, in turn, affects the observer's representation of the environmental states. The subtlety here is that such feedback is necessarily *delayed in time*. This means that at a given instant, only *previous* decisions can affect the observer's representation (e.g., through current sensory signals). Another instance of meta-Bayesian inference (which we have not explored here) that could couple the perceptual model to the response model is when the subject is observing his or herself (cf. meta-cognition).

The proposed meta-Bayesian procedure furnishes a generic statistical framework for (i) comparing different combinations of perceptual and response models and (ii) estimating the posterior distributions of their parameters. Effectively this allows us to make (approximate) Bayesian inferences about subject's Bayesian inferences. As stated in the introduction, the general IBDT problem is ill-posed; i.e. there are an infinite number of priors and loss-function pairs that could explain observed decisions. However, restricting the IBDT problem to estimating the parameters of a specific perceptual model (i.e. priors) and loss-function pair is not necessarily ill-posed. This is because the restricted IBDT problem can be framed as an inverse problem and finessed with priors (i.e., prior beliefs as an experimenter on the prior beliefs and loss-functions of a subject). As with all inverse problems, the identifiability of the BDT model parameters depends upon both the form of the model and the experimental design. This speaks to the utility of generative models for decision-making: the impact that their form and parameterisation has on posterior correlations can be identified before any data are acquired. Put simply, if two parameters affect the prediction of data in a similar way, their unique estimation will be less efficient.

Above, we noted (equation 12) that estimates of the subject's uncertainty might be inflated by experimental uncertainty. This may seem undesirable, as it implies a failure of veridical inference about subjects' beliefs (uncertainty). However, this non-trivial property is a direct consequence of optimal meta-Bayesian inference. The following example may illustrate how experimental uncertainty induces uncertainty about the subject's representation:

Say we know that the subject has a posterior belief that, with 90% confidence, some hidden state 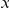 lies within an interval 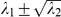, where 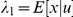 is their representation of 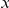
_,_ and 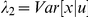 is the perceptual uncertainty. Now, we perform an experiment, measure behavioural responses 
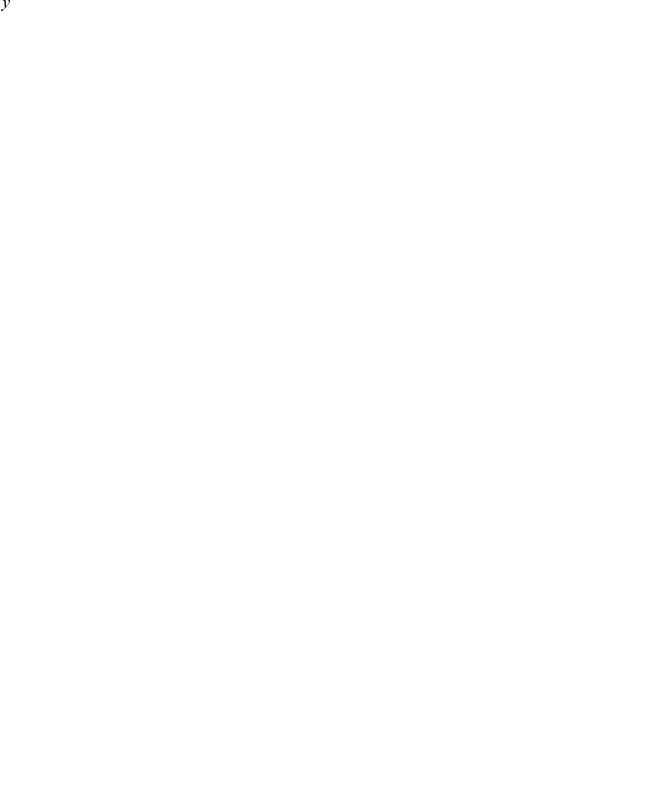
, and estimate 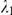 to lie within the credible interval 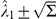, where 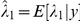 is our experimental estimate of 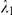 and 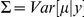 measures our experimental uncertainty about it. Then, our estimate of the subject's credible interval, when accounting for our experimental uncertainty about 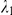, is 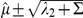. In general, this means that estimates of the subject's uncertainty are upper bounds on their actual uncertainty and these bounds become tighter with more precise empirical measures.

Finally, it is worth highlighting the importance of experimental design for identifying (Bayesian decision theoretic) models. This is because perceptual inference results from interplay between the history of inputs and the subject's priors. This means that an experimenter can manipulate the belief of the observer (e.g., creating illusions or placebo effects) and ensure the model parameters can be quantified efficiently. In our example, the identifiability of the perceptual and response parameters is determined by the magnitude of the sensory signals. This can then be optimized as part of the experimental design. More generally, the experimental design could itself be optimized in the sense of maximising sensitivity to the class of priors to be disclosed. In general, one may think of this as optimizing the experimental design for model comparison, which can be done by maximizing the discriminability of different candidate models ([67]).

In summary, the approach outlined in this paper provides a principled way to compare different priors and loss-functions through model selection and to assess how they might influence perception, learning and decision-making empirically. In a companion paper [68], we describe a concrete implementation of it and demonstrate its utility by applying it to simulated and real reaction time data from an associative learning task.

## Supporting Information

Appendix S1 Appendix S1 (‘*the variational Bayesian approach*’) is included as ‘supplementary material’. It summarizes the mathematical details of variational approximation to Bayesian inference under the Laplace approximation.
(DOC)

## References

[1] Daw ND, O'Doherty JP, Dayan P, Seymour B, Dolan RJ (2006). Cortical substrates for exploratory decisions in humans.. Nature. 2006 Jun 15;.

[2] Fehr E, Schmidt KM (1999). A theory of fairness, competition, and cooperation.. The Quarterly Journal of Economics.

[3] Kahneman D, Tversky A (1979). Prospect theory: An analysis of decisions under risk.. Econometrica.

[4] Beck JM, Ma WJ, Kiani R, Hanks T, Churchland AK (2008). Probabilistic population codes for Bayesian decision making.. Neuron.

[5] Dayan P, Hinton GE, Neal RM, Zemel RS (1995). The Helmholtz machine.. Neural Comput.

[6] Rao RP, Ballard DH (1999). Predictive coding in the visual cortex: a functional interpretation of some extra-classical receptive field effects.. Nat Neurosci.

[7] Friston K, Kilner J, Harrison L (2006). A free-energy principle for the brain,. J of physiol Paris.

[8] Helmholtz H (1925). Physiological optics, Vol. III: the perception of vision (J.P. Southall, Trans.),. Optical Soc. Of Am.

[9] Mach E (1980). Contributions to the analysis of the sensations (C. M. Williams, Trans.),.

[10] Kersten D, Knill DC, Mamassian P, Bülthoff I (1996). Illusory motion from shadows,. Nature.

[11] Rao RP (1998). An optimal estimation approach to visual perception and learning,. Vision Res.

[12] Lee TS (2003). Computations in the early visual cortex,. J Physiol Paris.

[13] Lee TS, Mumford D (2003). Hierarchical Bayesian inference in the visual cortex,. J Opt Soc Am A Opt Image Sci Vis.

[14] Mamassian P, Landy MS, Maloney MS, Rao R, Olshausen B, Lewicki M (2002). Bayesian modelling of visual perception. Probabilistic Models of the Brain.

[15] Mamassian P, Jentsch I, Bacon BA, Schweinberger SR (2003). Neural correlates of shape from shading,. NeuroReport.

[16] Weiss Y, Simoncelli EP, Adelson EH (2002). Motion illusions as optimal percepts,. Nature Neuroscience.

[17] Knill DC (1998). Discrimination of planar surface slant from texture: human and ideal observers compared.. Vision Res.

[18] Knill DC, Pouget A (2004). The Bayesian brain: the role of uncertainty in neural coding and computation,. Trends in Neurosci.

[19] Ernst MO, Banks MS (2002). Humans integrate visual and haptic information in a statistically optimal fashion,. Nature.

[20] Van Ee R (2003). Bayesian modelling of cue interaction: bistability in stereoscopic slant perception.. J Opt Soc Opt Am A Opt Image Sci Vis.

[21] Kording KP, Beierholm U, Ma WJ, Quartz S, Tenenbaum JB (2007). Causal inference in multisensory perception.. PLoS ONE.

[22] Wolpert DM (1995). An internal model for sensorimotor integration.. Science.

[23] Harris CM, Wolpert DM (1998). Signal-dependent noise determines motor planning.. Nature.

[24] Van Beers RJ (2002). Role of uncertainty in sensorimotor control.. Philos Trans R Soc Lond B Biol Sci.

[25] Trommershauser J, Maloney LT, Landy MS (2003). Statistical decision theory and trade-offs in the control of motor response,. Spatial vision.

[26] Kording KP, Fukunaga I, Howard IS, Ingram JN, Wolpert DM (2004). A neuroeconomics approach to inferring utility functions in sensorimotor control.. PloS Biol.

[27] Saunders JA, Knill DC (2004). Visual feedback control of hand movement.. J Neurosci.

[28] Behrens TE, Woolrich MW, Walton ME, Rushworth MF Learning the value of information in an uncertain world.. Nat Neurosci. 2007 Sep;.

[29] Den Ouden HEM, Daunizeau J, Roiser J, Friston KJ, Stephan KE (2010). Striatal prediction error modulates cortical coupling.. J Neurosci.

[30] Dayan P, Kakade S, Montague PR (2000). Learning and selective attention,. Nat Rev Neurosci.

[31] Dayan P, Yu AJ (2003). Uncertainty and learning,. IETE J Research.

[32] Tenenbaum JB, Kemp C, Shafto P, Feeney A, Heit E (2007). Theory-based Bayesian models of inductive reasoning.. Inductive reasoning.

[33] Tenenbaum JB, Griffiths TL, Kemp C (2006). Theory-based Bayesian models of inductive learning and reasoning.. Trends in Cognitive Sciences.

[34] Robert C (1992). L'analyse statistique Bayesienne..

[35] Von Neumann J, Morgenstern O (1944). Theory of games and economic behavior,.

[36] Hadamard J (1902). Sur les problèmes aux dérivées partielles et leur signification physique.. Princeton University Bulletin.

[37] Swartz RJ, Cox DD, Scott SB, Davies K, Follen M (2006). inverse decision theory: characterizing losses for a decision rule with applications in cervical cancer screening.. J am Stat Assoc.

[38] Wolpert D, Tumer K, Parsons S, Gmytrasiewicz G (2001). Reinforcement learning in distributed domains: an inverse game theoretic approach.. 2001 AAAI Spring Symposium on Game theoretic and decision theoretic agents.

[39] Ng AY, Russel S (2000). Algorithms for inverse reinforcement learning.. Proc 17^th^ Int Conf. Machine Learning.

[40] Abbeel P, Ng AY (2004). Apprenticeship learning via inverse reinforcement learning.. Proc 21^st^ Int Conf.

[41] Ramachandran D, Amir E (2007). Bayeisan inverse reinforcement learning.. Proc Conf. IJCAI-07.

[42] Krishnamurthy D, Todorov E (2009). Efficient algorithms for inverse optimal control.. Submitted.

[43] Beal M (2003). Variational algorithms for approximate Bayesian inference,.

[44] Brodersen KH, Penny WD, Harrison LM, Daunizeau J, Ruff CC (2008). Integrated Bayesian models of learning and decision making for saccadic eye movements.. Neural Networks.

[45] Spiegelhalter DJ, Best NG, Carlin BP, Van Der Linde A (2002). Bayesian measures of complexity and fit,. J R Statist Soc B.

[46] Friston K, Mattout J, Trujillo-Barreto N, Ashburner J, Penny W (2007). Variational free-energy and the Laplace approximation.. NeuroImage.

[47] Gold JI, Shadlen MN (2001). Neural computations that underlie decisions about sensory stimuli.. Trends Cogn Sci.

[48] Glimcher PW (2003). The neurobiology of visual-saccadic decision making.. Annu Rev Neurosci 2003;.

[49] Carpenter RH, Williams ML (1995). Neural computation of log likelihood in control of saccadic eye movements.. Nature.

[50] Frith U, Frith CD (2003). Development and neurophysiology of mentalizing.. Philos Trans R Soc Lond B Biol Sci.

[51] Yoshida W, Dolan RJ, Friston KJ (2008). Game Theory of Mind.. PLoS Comput Biol.

[52] Kersten D, Mamassian P, Yuille A (2004). Object perception as Bayesian inference,. Annual Review of Psychology.

[53] Kiebel SJ, Daunizeau J, Friston KJ (2009). Perception and hierarchical dynamics.. Frontiers in neuroinformatics (2009).

[54] Dayan P (2009). Dopamine, reinforcement learning, and addiction.. Pharmacopsychiatry.

[55] Dayan P, Huys QJM (2009). Serotonin in Affective Control.. Annual Review of Neuroscience.

[56] Yu AJ, Dayan P (2002). Acetylcholine in cortical inference.. Neural Networks.

[57] Dayan P (2009). Goal-directed control and its antipodes.. Neural Networks.

[58] Friston KJ, Daunizeau J, Kiebel SJ (2009). Reinforcement learning or active inference.. Plos ONE.

[59] Friston KJ, Daunizeau J, Kilner J, Kiebel SJ (2010). Action and behaviour: a free energy formulation.. Bio Cybern.

[60] Nienborg H, Cumming BG (2009). Decision-related activity in sensory neurons reflects more than a neuron's causal effect.. Nature.

[61] Kirk DE (2004). Optimal control theory: an introduction..

[62] Shoham Y, Leyton-Brown K (2009). Multiagent Systems: Algorithmic, Game-Theoretic, and Logical Foundations,.

[63] Sutton RS, Barto AG (1998). Reinforcement learning: an introduction,.

[64] Todorov E, Scholkopf (2006). Linearly-solvable Markov decision problems.. In Advances in Neural Information Processing Systems.

[65] Watkins CJCH, Dayan P (1992). Q-learning.. Machine Learning.

[66] McClelland JL, Botvinick MM, Noelle DC, Plaut DC, Rogers TT (2010). Letting Structure Emerge: Connectionist and Dynamical Systems Approaches to Understanding Cognition.. Trends in Cognitive Sciences.

[67] Daunizeau J, Preuschoff K, Friston KJ, Stephan KE (in preparation). Optimizing experimental design for Bayesian model comparison.

[68] Daunizeau J, Den Ouden HEM, Pessiglione M, Kiebel SJ, Friston KJ (2010). Observing the Observer (II): Deciding when to decide.. PLoS ONE.

