# Supplementary material for: Observing the Observer (I): Meta-Bayesian Models of Learning and Decision-Making
Source: PLoS One. 2010 Dec 14;5(12):e15554. doi: 10.1371/journal.pone.0015554 (PMC3001878; doi:10.1371/journal.pone.0015554)
Supplement: Appendix S1 — (‘the variational Bayesian approach’) is included as ‘supplementary material’. It summarizes the mathematical details of variational approximation to Bayesian inference under the Laplace approximation. (DOC) [file pone.0015554.s001.doc]

**Appendix S1 (OTO I): the variational Bayesian approach**

In this appendix, we summarize the variational Bayesian approach, which is used both to model the recognition process (i.e. the subject’s inversion of the perceptual model), and to invert the response model.

In this appendix, $u$ denotes the data, $x$ its hidden causes and $m$ the generative model (we dropped any time/trial index for simplicity). Let us recall that the model evidence is lower bounded by the so-called free energy $F\left(q,u\right)$, where $q\left(x\right)$ is any probability density function over $x$:

$\begin{matrix} F\left(q,u\right)=\ln p\left(u\left|m\right.\right)-D \\ =U-S \\ D=\int q\left(x\right)\ln\frac{q\left(x\right)}{p\left(x\left|u,m\right.\right)}dx\geq0 \end{matrix}$. A1

The free-energy comprises an energy term $U=\left\langle\ln p\left(x,u\left|m\right.\right)\right\rangle_{q}$ and an entropy term $S=\left\langle\ln q\left(x\right)\right\rangle_{q}$, where denotes the expectation under $q$. Note that all these quantities are the negative of their thermodynamic homologues. The free-energy is a lower bound on the log-evidence because the Kullback-Leibler cross=entropy or divergence $D$ between the arbitrary and posterior densities is non-negative. Maximizing the free-energy with respect to $q\left(x\right)$ minimizes the divergence, rendering the arbitrary density $q\left(x\right)\approx p\left(x\left|u,m\right.\right)$ an approximate posterior density.

To make this approximation easier, one usually assumes $q\left(x\right)$ factorizes into approximate marginal posterior densities, over sets of hidden causes $x_{i}$:

$q\left(x\right)=\prod_{i} q_{i}\left(x_{i}\right)$. A2

In statistical physics, this is called a mean-field approximation (see .e.g. Yedidia 2000 for a machine-learning perspective on mean-field theory). This approximation replaces stochastic dependencies between the partitioned hidden causes by deterministic relationships between the sufficient statistics of their approximate marginal posterior density (see below). Under the mean-field approximation, it is straightforward to show that the approximate marginal posterior densities satisfy the following set of equations:

$\begin{matrix} \frac{\delta F}{\delta q}=0\Rightarrow q_{i}\left(x_{i}\left|\lambda_{i}\right.\right)=\frac{1}{Z_{i}}\exp\left(I\left(x_{i}\right)\right) \\ I\left(x_{i}\right)=\int\prod_{j\neq i} dx_{j}q_{j}\left(x_{j}\left|\lambda_{i}\right.\right)\ln p\left(x,u\left|m\right.\right) \end{matrix}$, A3

where $\lambda_{i}$ are the sufficient statistics of the approximate marginal posterior density $q_{i}$, and $Z_{i}$ is a normalization constant (i.e., the partition function). We will call $I\left(x_{i}\right)$ the variational energy. If the integral in eq. A3 is analytically tractable (e.g., through the use of conjugate priors) the above Boltzmann equation provides us with a closed form update rule for the sufficient statistics. Iterating these updates then optimizes the free energy with respect to the approximate posterior density. The ensuing variational Bayesian schemes subsume many other common inference schemes as special cases (e.g. maximum-likelihood, maximum *a posteriori*, expectation-maximization; see Beal 2003).

When inverting realistic generative models, nonlinearities in the likelihood function generally induce posterior densities that are not in the conjugate-exponential family. This means that there are an infinite number of sufficient statistics of the approximate posterior density; rendering the integral in eq; A3 analytically intractable. The *Laplace approximation* is a useful and generic device, which can finesse this problem by reducing the set of sufficient statistics of the approximate posterior density to its first two moments. This means that each approximate marginal posterior density is further approximated by a Gaussian density:

$q_{i}\left(x_{i}\left|\lambda_{i}\right.\right)=N\left(\lambda_{i}\right):\lambda_{i}=\left(\begin{matrix} \mu_{i}=\left\langle x_{i}\right\rangle\\ \Sigma_{i}=\left\langle\left(x_{i}-\mu_{i}\right)\left(x_{i}-\mu_{i}\right)^{T}\right\rangle\end{matrix}\right)$ A4

where the sufficient statistics $\lambda_{i}=\left\{\mu_{i},\Sigma_{i}\right\}$ encode the posterior mean and covariance of the $i$-th approximate marginal posterior density. This (fixed-form) Gaussian approximation is derived from a second-order Taylor expansion to the variational energy:

$\begin{matrix} \mu_{i}=\mathop{\mathrm{argmax}}\limits_{x_{i}} I\left(x_{i}\right) \\ \Sigma_{i}=-\left[\left.\frac{\partial^{2}L}{\partial x_{i}^{2}}\right|_{\mu_{i}}\right]^{-1} \\ I\left(x_{i}\right)\approx L\left(x_{i},\mu_{\neg i}\right)+\sum_{j\neq i} tr\left[\left.\frac{\partial^{2}L}{\partial x_{i}^{2}}\right|_{\mu}\Sigma_{j}\right] \\ L\left(x\right)=\ln p\left(x,u\left|m\right.\right) \end{matrix}$. A5

Equation A5 defines each variational energy and approximate marginal posterior density as explicit functions of the sufficient statistics of the other approximate marginal posterior densities. Under the VB-Laplace approximation, the iterative update of the sufficient statistics just requires the gradients and curvatures of the log-joint density $\ln p\left(x,u\left|m\right.\right)$ with respect to the hidden causes. Thus, when the Laplace approximation is used on all mean-field partitions, the free energy can then be evaluated as follows:

$F=L\left(\mu\right)+\frac{1}{2}\sum_{i} \left(\ln\left|\Sigma_{i}\right|+p_{i}\ln2\pi\right)$, A6

where $p_{i}$ is the dimension of the $i$-th partition. This makes it a very quick and robust approximate Bayesian inference approach (see Friston et al. 2007). In our context, we suggest to use such a VB-Laplace scheme both when deriving the recognition process and when inverting the response model.

**References**

Beal M. (2003), *Variational algorithms for approximate Bayesian inference*, PhD thesis, ION, UCL, UK.

Friston K., Mattout J., Trujillo-Barreto N., Ashburner J., Penny W. (2007), *Variational free-energy and the Laplace approximation*. NeuroImage 1: 220-234.

Yedidia J. S. (2000) *An idiosyncratic journey beyond mean-field theory*. MIT Press, Math6X9/2000/06.
